# TiSi_2_ Phase Formation in Magnetron-Sputtered Ti Films on Silicon During Rapid Thermal Annealing

**DOI:** 10.3390/nano16191258

**Published:** 2026-10-06

**Authors:** Symaiyl Keiinbay, Kair Kh. Nussupov, Nurzhan B. Beisenkhanov, Alex A. Volinsky

**Affiliations:** 1 School of Materials Science and Green Technologies, Kazakh-British Technical University, Tole bi 59, Almaty 050000, Kazakhstan; s.keiinbay@kbtu.kz (S.K.); k.nusupov@kbtu.kz (K.K.N.); 2 Department of Mechanical and Aerospace Engineering, University of South Florida, 4202 E. Fowler Ave. ENG030, Tampa, FL 33620, USA

**Keywords:** titanium disilicide, TiSi_2_, C54 phase, magnetron sputtering, rapid thermal annealing, sheet resistance, X-ray diffraction, thin film, silicon, silicidation

## Abstract

Titanium thin films were deposited on crystalline silicon substrates by DC magnetron sputtering and annealed by rapid thermal processing under vacuum and nitrogen environments. X-ray diffraction, atomic force microscopy, and four-point probe measurements were used to evaluate phase formation and electrical properties. Annealing at 600 °C for 5 min in 10^−3^ Pa high vacuum produces the C54-TiSi_2_ phase, reducing sheet resistance from 56 Ω/sq to 1.94 Ω/sq. Lowering the synthesis temperature from 923 K to 873 K decreases radiative energy consumption by approximately 20%. In contrast, annealing in N_2_ atmosphere yields Ti_5_Si_4_ and TiN instead of TiSi_2_. A combined thermodynamic and diffusion model explains the observed phase selectivity under vacuum and nitrogen environments.

## 1. Introduction

The continuous scaling of semiconductor devices in accordance with Moore’s law demands contact materials that simultaneously satisfy requirements of high electrical conductivity, chemical compatibility with silicon, and long-term thermal stability [1,2]. Among refractory metal silicides, titanium disilicide TiSi_2_ has long been recognized as one of the most promising candidates for self-aligned silicide (SALICIDE) contacts in complementary metal-oxide-semiconductor (CMOS) technology. Its low bulk resistivity, compatibility with both n-type and p-type silicon, and stability at elevated processing temperatures make it particularly attractive for sub-micron device architectures [1,3].

TiSi_2_ is a polymorphic material that can exist in two crystallographically distinct orthorhombic phases. The metastable C49 phase (base-centered orthorhombic, space group Cmcm) forms preferentially at lower annealing temperatures and exhibits a relatively high resistivity in the range of 60 to 90 μΩ·cm. Upon annealing at higher temperatures, the C49 phase undergoes a reconstructive transformation to the thermodynamically stable C54 phase (face-centered orthorhombic, space group Fddd), which has a substantially lower resistivity [2,4]. Achieving direct C54 formation at the lowest possible thermal budget is a central challenge in modern silicidation research.

Recent advances in physical vapor deposition have significantly improved control over TiSi_2_ thin film morphology and crystallinity. Optimization of magnetron sputtering parameters, including power, working pressure, and substrate temperature, enables nanometer-level control of film thickness and enables synthesis suitable for both CMOS and microelectromechanical systems (MEMS) applications [3,5]. Novel approaches such as room-temperature reactive sputtering have further broadened the synthesis of TiSi-based compounds, including TiSiN coatings with enhanced hardness and oxidation resistance [6].

The conventionally reported temperature window for the C49 to C54 transition lies between 700 and 900 °C, primarily for Ti films reacting on bulk crystalline silicon substrates, where microstructure constraints increase the transformation barrier [4,7,8,9]. This distinction is important because the present work employs sputtered nanocrystalline titanium, whose high grain boundary density enables C54 formation at substantially lower temperatures. Recent work demonstrates that nanosecond laser annealing can induce C54 formation at lower effective temperatures by creating favorable microstructure templates during the laser pulse, thereby expanding the processing space for thermally sensitive device architectures [10]. Electromagnetic directional solidification studies have provided complementary theoretical insights into controlling the C49 to C54 phase transformation pathway [11].

Beyond microelectronics, TiSi_2_ thin films are increasingly used in MEMS sensors, where they enhance device sensitivity while enabling miniaturization [12,13]. The excellent high-temperature oxidation resistance and mechanical robustness of TiSi_2_ also make it a promising candidate for aerospace and defense coatings requiring lightweight and durable materials [14].

The role of the annealing atmosphere in determining the final phase composition has received comparatively less attention. In particular, the competitive formation of TiN and titanium sub-silicides (Ti_5_Si_4_) during annealing in nitrogen remains incompletely understood from a mechanistic standpoint, particularly regarding the quantitative roles of diffusion blocking versus kinetic competition for Ti atoms [15,16].

In the present work, Ti thin films were deposited on crystalline silicon (c-Si) wafers by DC magnetron sputtering and subjected to rapid thermal annealing under two distinct atmospheres, high vacuum and nitrogen. The structural evolution and electrophysical properties were characterized by X-ray diffraction (XRD), atomic force microscopy (AFM), and four-point probe measurements. A thermodynamic and diffusion model was developed to provide a mechanistic framework for experimental observations, with emphasis on the surprisingly low C54 formation temperature and the atmosphere-dependent phase selectivity.

## 2. Materials and Methods

### 2.1. Sample Preparation

Crystalline silicon wafers were used as substrates. Prior to deposition, three-stage chemical cleaning was performed to ensure surface purity. Ti thin films were deposited by DC magnetron sputtering under the following conditions: 300 W power, 0.9 Pa working pressure, and 16.6 cm^3^/min Ar flow rate. Ti and TiN 100 mm diameter sputtering targets (99.95% pure) were used for deposition at a working distance of 100 mm. Ti films with a nominal thickness of 40 nm were deposited in 126 s, while 50 nm films were deposited in 158 s. TiN capping layers (10 nm, 29 s) were deposited at 1200 W with an N_2_/Ar flow ratio of 30:1 (30 cm^3^/min N_2_:1 cm^3^/min Ar) at 0.8 Pa and a substrate temperature of 100 °C. The following Ti/TiN film systems were prepared: (i) Ti 50 nm (158 s), (ii) Ti 40 nm (126 s) and TiN 10 nm (29 s), and (ii) Ti 25 nm (79 s) and TiN 25 nm (72 s).

### 2.2. Rapid Thermal Annealing

Samples were annealed in two furnace systems. The first system, an GS-TF-1200 tube furnace (Shenzhen Gauss Scientific Instrument Co., Ltd., Shenzhen, China) operating at 7 to 8 Pa, was used for initial annealing trials at 600 to 800 °C for 5 min. This furnace operates with a heating rate of approximately 5 °C/min. The second system, MAGNA TM-200-01 furnace (NIITM, Research Institute of Precision Engineering, Zelenograd, Russia) capable of achieving 10^−3^ Pa vacuum or a controlled nitrogen atmosphere, was used for annealing at 600 to 750 °C in vacuum and at 720 °C in N_2_ for 1 to 5 min. This system provides a significantly higher heating rate of approximately 10 °C/sec, which can influence phase formation kinetics and contributes to the low-temperature C54 phase formation observed in this work.

### 2.3. Characterization Methods

Phase composition and crystallographic structure were determined by X-ray diffraction using CuKα radiation. Surface morphology was analyzed by atomic force microscopy. Sheet resistance was measured at five points per sample using the four-point probe method to ensure statistical reliability.

## 3. Results

### 3.1. TiO_2_ Formation in the SP1200 Tube Furnace

**Figure 1:**
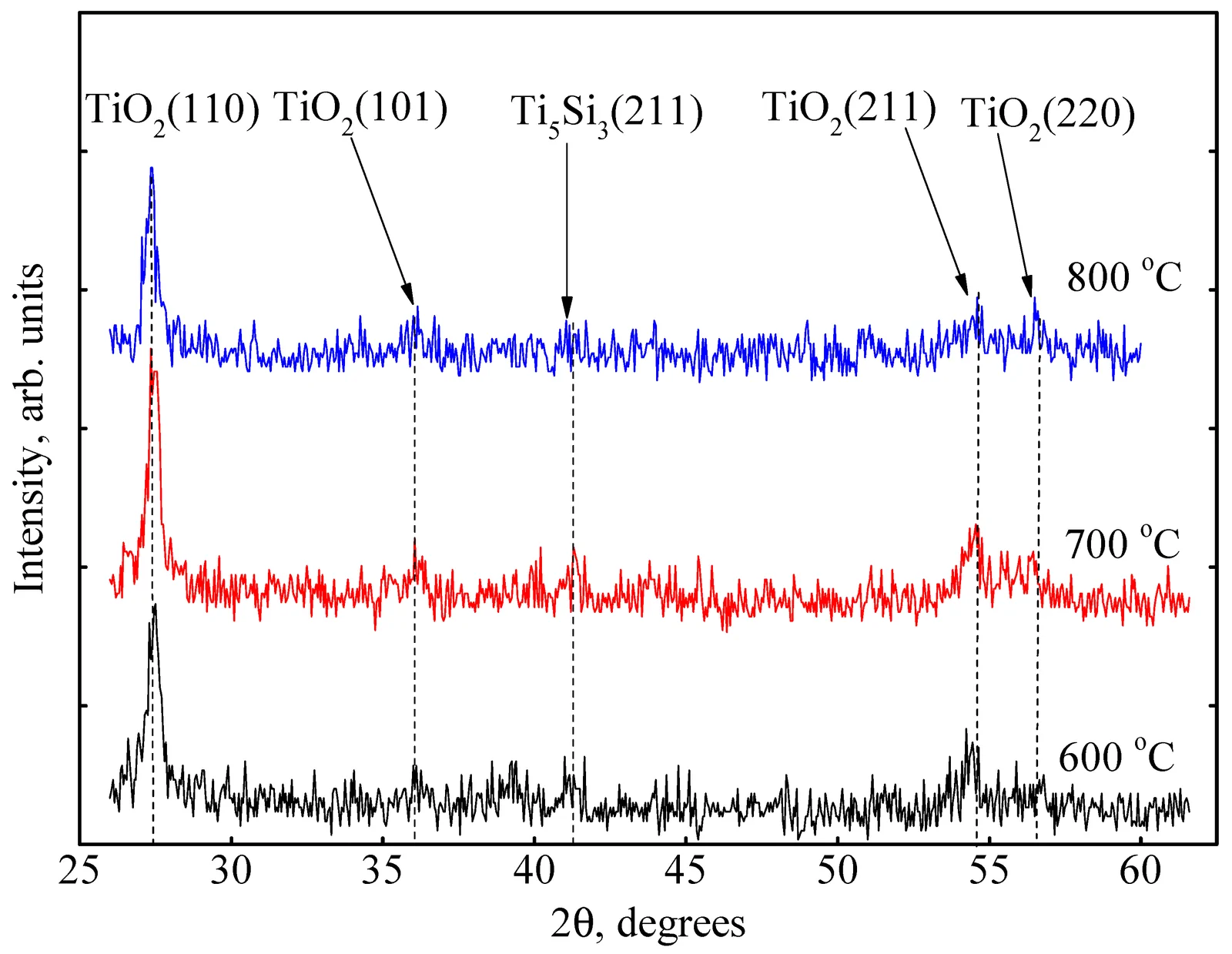
XRD patterns of c-Si/Ti 50 nm structures after annealing in the SP1200 tube furnace at 7–8 Pa for 5 min at 600, 700, and 800 °C.

Annealing in the SP1200 furnace at a residual pressure of 7 to 8 Pa led to complete oxidation of the titanium films. XRD analysis showed the disappearance of the metallic Ti phase and the appearance of TiO_2_ in the rutile structure in Figure 1. Sheet resistance values in the range of 70 to 90 Ω/sq confirmed oxide formation rather than silicidation. These results are consistent with the known high affinity of titanium for oxygen at elevated temperatures. Even trace amounts of residual oxygen at multi-pascal pressures are sufficient to suppress Si-Ti interdiffusion and drive the system toward oxide formation [2]. This result underscores the critical importance of achieving 10^−3^ Pa vacuum or better for silicidation applications.

Figure 1 presents XRD patterns collected from Ti films annealed at 600, 700, and 800 °C. In all three cases, characteristic rutile TiO_2_ reflections are observed, including the (110) at ~27.4° and the (101) at ~36.1° 2θ, whose intensities increase with annealing temperature, indicating progressive oxidation and grain growth. No silicide reflections are detected. These results conclusively demonstrate that annealing at pressures above approximately 1 Pa is incompatible with silicidation, as residual oxygen preferentially oxidizes titanium before Si-Ti interdiffusion can proceed.

### 3.2. C54-TiSi_2_ Formation Under High Vacuum

Annealing of Ti films in the TM-200-01 furnace at 10^−3^ Pa vacuum in the temperature range of 600 to 750 °C for 5 min produced distinct TiSi_2_ reflections in the XRD patterns. Diffraction reflections at 2θ values of 39.31, 42.4, and 50.07° correspond to the (311), (004), and (313) reflections of the C54-TiSi_2_ phase (face-centered orthorhombic, space group Fddd), with lattice parameters a = 8.24 Å, b = 4.78 Å, and c = 8.54 Å. The corresponding sheet resistance decreased dramatically from a pre-annealing value of 56 Ω/sq to 1.94 Ω/sq after annealing at 600 °C, representing approximately a 30-fold reduction.

**Figure 2:**
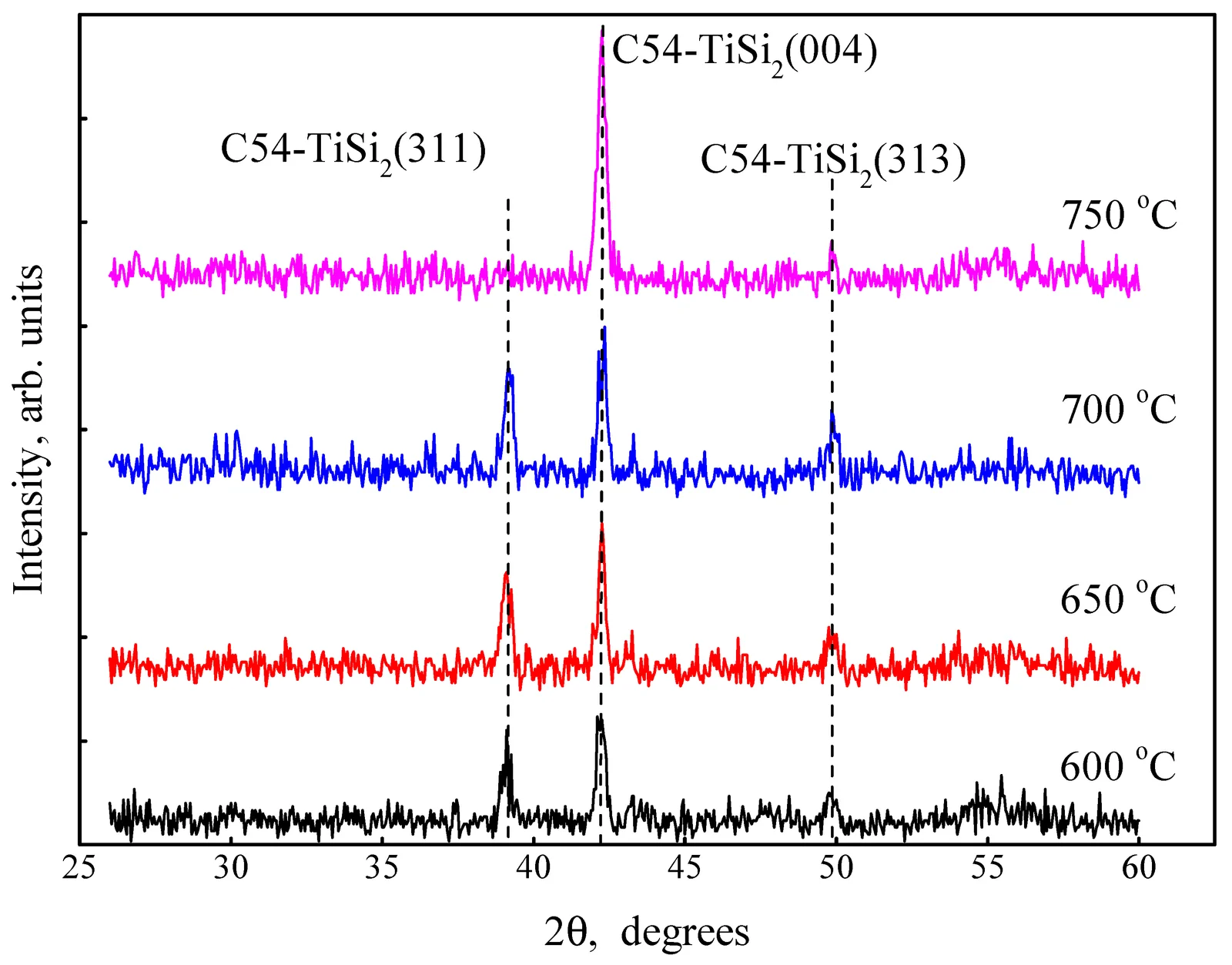
XRD patterns of TiSi_2_ structures formed after annealing 50 nm Ti film on c-Si in the AS-One 150 furnace under 10^−3^ Pa vacuum for 5 min at 600, 650, 700, and 750 °C.

Maximum XRD intensity positions in Figure 2 correspond to C54-TiSi_2_ (311), (004), and (313) reflections. At 750 °C, the (311) reflection disappears and the (004) intensity increases, indicating preferential crystal orientation (texture development). As seen in Figure 2, C54-TiSi_2_ forms at all four annealing temperatures, including as low as 600 °C. At 600 °C, broad nanocrystalline reflections are observed, consistent with small coherent scattering domains. Increasing the temperature to 650 °C leads to increased intensities, reflecting a greater volume fraction of C54 grains. At 700 °C, reflection widths narrow further, indicating crystallite growth.

The observation of C54-TiSi_2_ at 600 °C is notable. Conventional single-step rapid thermal annealing (RTA) of Ti on bulk silicon typically requires temperatures of 700 to 900 °C for the C49-to-C54 transition, with the C49 phase forming as an intermediate between approximately 500 and 700 °C [4,7]. The direct formation of C54 at 600 °C observed here is attributed to the nanocrystalline character of the sputtered Ti films and the exceptionally clean thermodynamic environment maintained by the 10^−3^ Pa vacuum, as discussed further in Section 4.

### 3.3. Sheet Resistance Measurements

**Figure 3:**
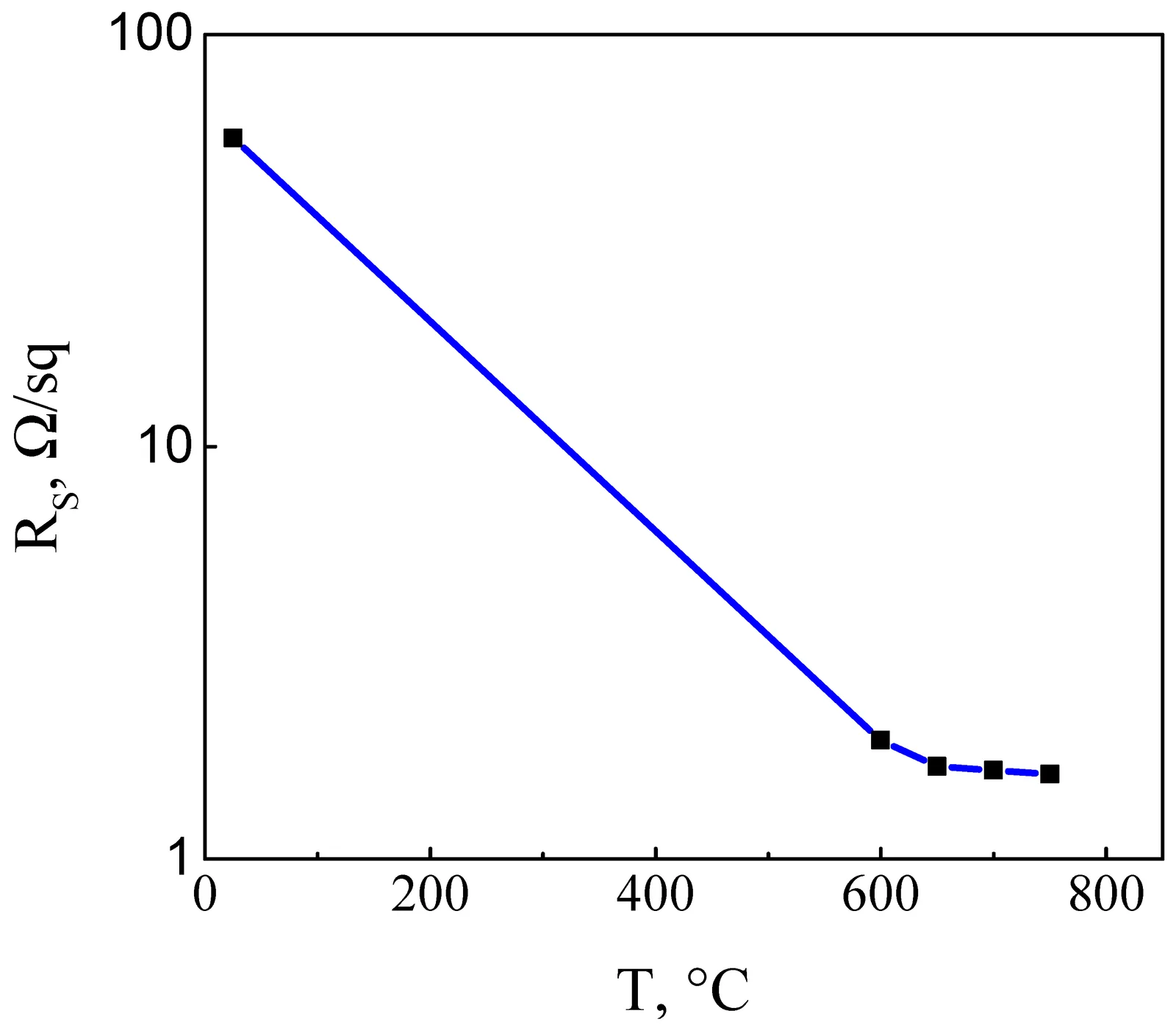
Sheet resistance R_s_ of as-deposited 50 nm Ti films on c-Si and as a function of RTA temperature.

Figure 3 illustrates the sheet resistance R_s_ evolution with annealing temperature. As-deposited Ti films exhibit R_s_ value of 56 Ω/sq, confirming uniform and well-controlled deposition conditions. Upon annealing at 600 °C, R_s_ drops sharply to 1.94 Ω/sq, a reduction of approximately 30-fold. This dramatic change is directly attributable to the transformation of resistive metallic Ti into the highly conductive C54-TiSi_2_ phase, as confirmed by XRD. The results are consistent with the known resistivity of C54-TiSi_2_ (12 to 24 μΩ·cm), compared with Ti films (approximately 60 μΩ·cm). Subsequent annealing at 650, 700, and 750 °C yields a gradual further decrease in R_s_, from 1.945 to 1.677, 1.641, and 1.605 Ω/sq, respectively. This monotonic decrease represents only a 1.21-fold reduction across 150 °C and is attributed to structural ordering and crystallite growth within the C54-TiSi_2_ phase rather than a new phase transition.

### 3.4. Ti/TiN Systems in N_2_ Atmosphere

Annealing of Ti/TiN bilayer systems in N_2_ atmosphere at 720 °C produced entirely different phase assemblages compared to those obtained in high vacuum. XRD analysis revealed the formation of Ti_5_Si_4_ sub-silicide and TiN nanocrystals rather than TiSi_2_.

**Figure 4:**
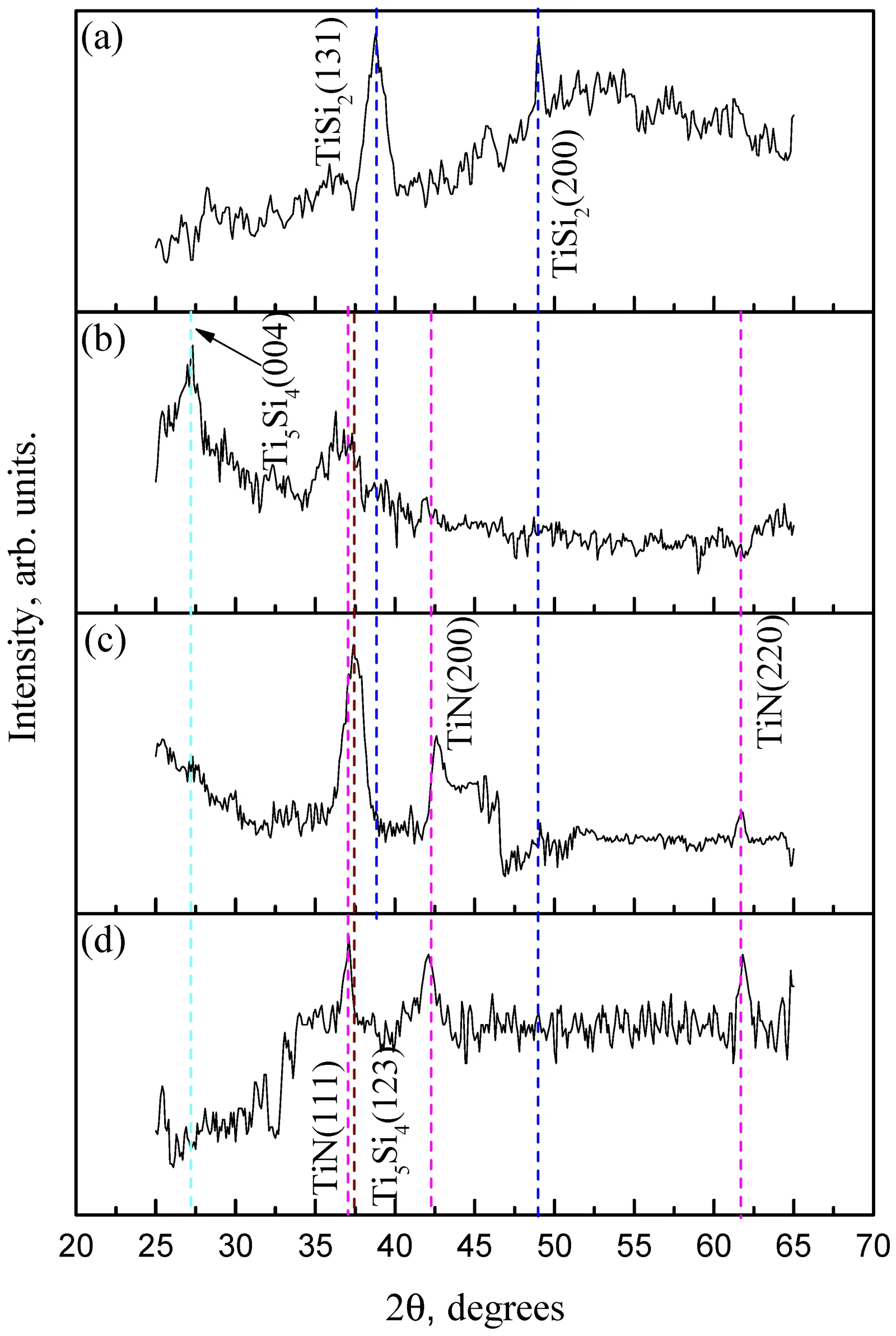
XRD patterns of Ti and TiN films on c-Si after rapid thermal annealing at 720 °C: (**a**) Ti (50 nm) for 5 min in vacuum; (**b**) Ti (40 nm) and TiN (10 nm) for 5 min in N_2_; (**c**) Ti (25 nm) and TiN (25 nm) for 1 min in N_2_; (**d**) Ti (25 nm) and TiN (25 nm) for 5 min in N_2_.

Figure 4 summarizes the phase evolution under different Ti/TiN stack configurations and annealing conditions. Figure 4a shows that in the Si/Ti (50 nm) system annealed in vacuum, TiSi_2_ (131) and (200) reflections are observed without any sub-silicide or nitride phases, consistent with Section 3.2. In contrast, Figure 4b–d demonstrate that when a TiN capping layer is present and annealing is performed in N_2_, the system produces Ti_5_Si_4_ and TiN rather than TiSi_2_. In the Si/Ti (40 nm)/TiN (10 nm) system in Figure 4b, Ti_5_Si_4_ (004) and (123) reflections are observed alongside TiN (111) reflections. The Si/Ti (25 nm)/TiN (25 nm) system annealed for 1 min in Figure 4c shows Ti_5_Si_4_ (123) and TiN (111), (200), and (220) reflections. Extending the annealing duration to 5 min in Figure 4d increases the TiN (200) reflection intensity relative to (111), indicating the development of a preferred TiN texture along the [200] direction and enhanced surface granulation, as corroborated by AFM.

**Figure 5:**
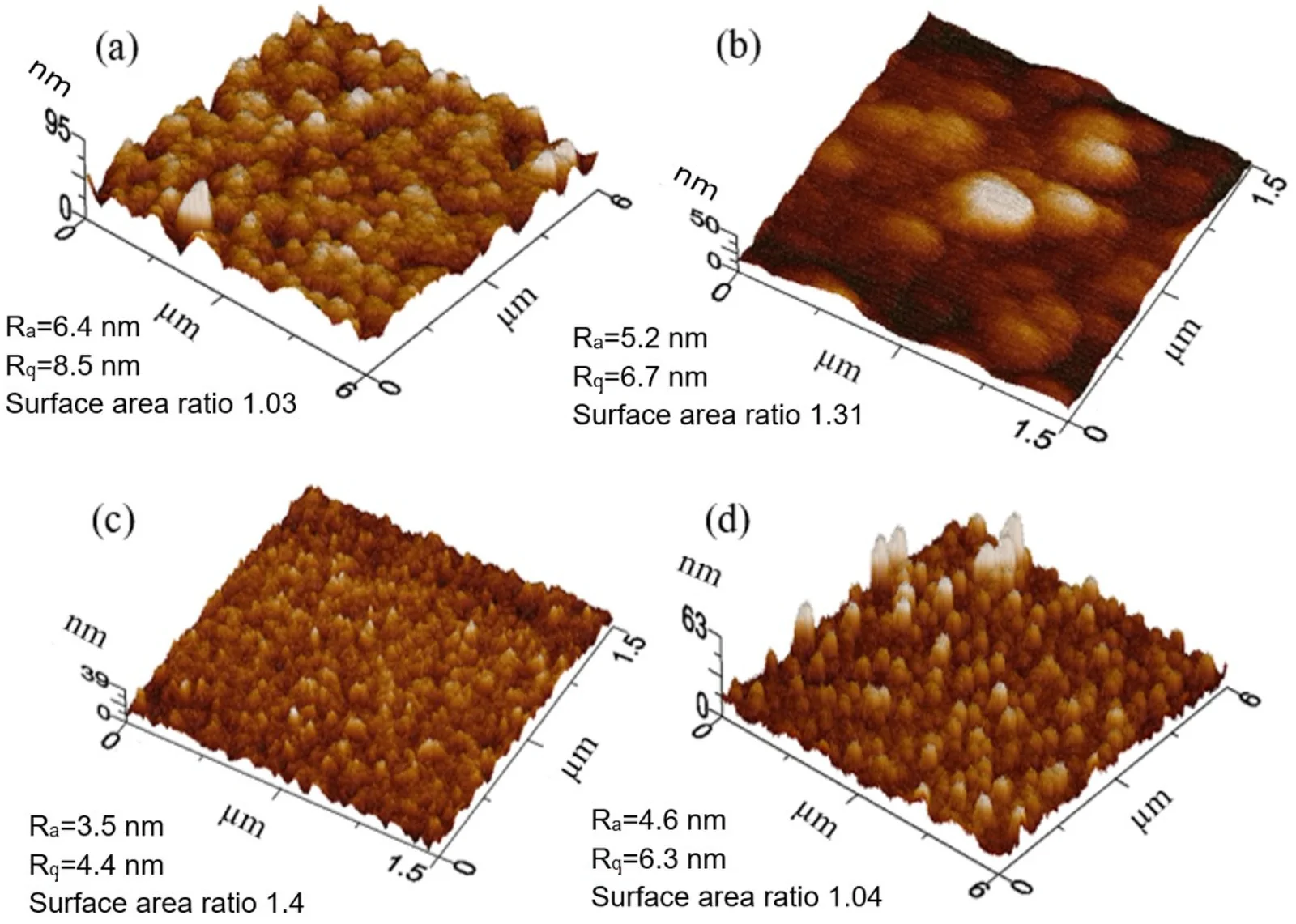
AFM surface morphology of Ti and TiN films on Si after RTA at 720 °C: (**a**) Ti (50 nm) in vacuum; (**b**) Ti (40 nm) and TiN (10 nm) in N_2_; (**c**) Ti (25 nm) and TiN (25 nm) in N_2_, 1 min; (**d**) Ti (25 nm) and TiN (25 nm) in N_2_, 5 min. Here, R_a_ is arithmetic average roughness, and R_q_ is root mean square roughness.

Progressive surface grain coarsening is observed with increasing TiN fraction and annealing duration, consistent with TiN texture development observed by XRD. AFM images in Figure 5 show clear changes in surface morphology across the four film systems. Figure 5a presents the TiSi_2_ film with a fine-grained surface and an RMS roughness Rq of 8.5 nm. Figure 5b shows the Ti_5_Si_4_ TiN film with larger surface features, an RMS roughness of 6.7 nm, and an increased surface area ratio. Figure 5c exhibits pronounced surface granulation as TiN thickness increases, together with a reduced RMS roughness of 4.4 nm and a surface area ratio of 1.4. Figure 5d shows the annealed film with an RMS roughness of 6.2 nm and a lower surface area ratio, consistent with TiN grain growth and partial reorganization of the surface relief. These morphological changes have direct implications for the electrical properties and reliability of contacts fabricated from these film systems.

## 4. Thermodynamic and Diffusion Model of TiSi_2_ Phase Formation

### 4.1. Stefan–Boltzmann Energy Framework

The thermal energy delivered to the film during RTA scales with the fourth power of absolute temperature according to the Stefan–Boltzmann relation for radiative power density

*P* = *σT*^4^ (1)

where *σ* = 5.67 × 10^−8^ W·m^−2^·K^−4^ is the Stefan–Boltzmann constant and *T* is the absolute temperature in Kelvin. Reducing the annealing temperature from *T*_1_ = 923 K (650 °C) to *T*_2_ = 873 K (600 °C) yields a fractional energy reduction

(2) $$\frac{\Delta P}{P}=1-{\left(\frac{T_{2}}{T_{1}}\right)}^{4}=1-{\left(\frac{873}{923}\right)}^{4}\approx 0.2$$

When referenced against the conventional C49-to-C54 transition temperature of *T*_ref_ = 973 K (700 °C), the energy reduction from 973 K to 873 K exceeds 35%. This is a technologically significant gain for industrial annealing equipment operating in continuous manufacturing environments, where cumulative thermal budgets directly affect throughput costs and substrate integrity [17].

### 4.2. Arrhenius Diffusion Model for Si-Ti Interdiffusion

The formation of TiSi_2_ requires interdiffusion of Si and Ti atoms across the film interface. The effective diffusion coefficient follows the Arrhenius relationship

(3) $$D\left(T\right)=D_{0}\cdot \exp\left(-\frac{E_{a}}{k_{B}T}\right)$$

where *D*_0_ is the pre-exponential factor, *E_a_* is the activation energy for Si-Ti interdiffusion with reported values of 1.5 to 2 eV [4], and *k_B_* is Boltzmann’s constant. The diffusion length *x* over annealing time *t* is given by

(4) $$x=2\sqrt{D(T)\cdot t}$$

For a 50 nm Ti film annealed at 600 °C (873 K) for 300 s with *E_a_* = 1.8 eV and *D*_0_ = 10^−4^ m^2^/s [18], the calculated diffusion coefficient *D*(*T*) ≈ 3.8 × 10^−15^ m^2^/s and diffusion length *x* ≈ 2 μm. This value substantially exceeds the 50 nm Ti film thickness, confirming that complete transformation of Ti into TiSi_2_ is feasible at 600 °C under high vacuum conditions.

### 4.3. Thermodynamic Phase Stability and Annealing Atmosphere Effects

To understand the competing phase formation pathways observed experimentally, we analyze the thermodynamic stability of C54-TiSi_2_, Ti_5_Si_4_, TiN, and rutile TiO_2_ phases using the Gibbs free energy of formation from the elements. The standard molar Gibbs free energy of formation Δ*G_f_* for each phase is computed from enthalpy Δ*H_f_* and entropy Δ*S_f_*

Δ*G_f_*=Δ*H_f_* − *T*⋅Δ*S_f_* (5)

Here, Δ*H_f_* denotes the enthalpy of formation, the standard enthalpy change for forming one mole of the compound from the constituent elements in their reference states at the same temperature and pressure. Similarly, Δ*S_f_* is the entropy of formation, equal to the molar entropy of the compound minus the stoichiometric sum of the molar entropies of the constituent elements, all evaluated at the specified temperature and standard pressure *p* = 0.1 MPa. These are intensive molar quantities with kJ/mol and J/(mol·K) units, respectively.

The negative Gibbs energy change (Δ*G_f_* < 0) indicates thermodynamic spontaneity for formation from the elements in their reference states, but does not uniquely determine which phase forms first, nor does it establish the kinetic rate of formation or which phase is preferred among competing alternatives. A more negative Δ*G_f_* implies a larger thermodynamic driving force. When Δ*H_f_* and Δ*S_f_* are both approximately constant with temperature, the term −*T*Δ*S_f_* increases in absolute magnitude with increasing temperature (becoming more negative if Δ*S_f_* is negative, or more positive if Δ*S_f_* is positive), causing Δ*G_f_* to become less negative as temperature increases in the former case.

Table 1 provides calculated thermodynamic quantities at three representative annealing temperatures used in this work (873, 923, 973, and 1023 K) drawn from published thermochemical databases, including Joint Army-Navy-Air Force Thermochemical Tables (JANAF) [19]. The thermochemical basis for the current analysis was cross-checked against several independent literature sources: the TiSi_2_ calorimetric measurements of Kematick and Myers [20], the CALculation of PHAse Diagrams (CALPHAD) thermodynamic assessment of Fiore et al. [21], the high-temperature direct calorimetry of Topor and Kleppa [22], the critical evaluation and assessment of Chandrasekharaiah et al. [23], and Sylla et al. [24]. Individual determinations from different sources were not averaged or combined into a single fitted dataset. Instead, one selected model was retained for each compound to maintain thermodynamic consistency.

### 4.4. Thermodynamic Analysis of TiSi_2_ and TiN Formation

Based on multiple sources and Table 1, Δ*G_f_*(TiSi_2_) does not change significantly over the 873–1023 K temperature range. These results confirm that TiSi_2_ formation from the constituent elements is thermodynamically favorable. However, the magnitude of Δ*G_f_* alone does not distinguish between the C49 and C54 polymorphic phases, nor does it predict whether direct C54 nucleation can occur without an intermediate C49 stage. These questions require kinetic and microstructure considerations.

In nitrogen-containing atmospheres, TiN formation competes with TiSi_2_ formation. The relevant comparison is the standard Gibbs energy for the reaction

TiSi_2_ + ½N_2_ → TiN + 2Si (6)

TiN formation is thermodynamically favorable relative to TiSi_2_. For pure solids and ideal nitrogen gas

(7) $$\Delta G_{f}=\Delta G_{f}^{\circ }-\frac{\mathrm{RT}}{2}\ln\left(\frac{p_{N_{2}}}{p^{\circ }}\right)$$

Here, the natural logarithmic term accounts for the partial pressure of N_2_ relative to the standard pressure *p* = 0.1 MPa. The actual nitrogen partial pressure during annealing and the relative kinetic rates of TiN formation versus Si–Ti interdiffusion are thus required to translate thermodynamic driving force into a mechanistic prediction of which phases form.

It is important to note that nitrogen exposure does not universally eliminate TiSi_2_ under all conditions. Desu and Taylor [25] observed TiN layers forming above 700 °C over C54-TiSi_2_ in nitrogen atmosphere, but direct nitridation of pre-formed TiSi_2_ (conversion of the silicide to the nitride) did not become significant until temperatures exceeded 950 °C. This distinction between competing formation pathways and post-formation reaction pathways is essential. Thermodynamic driving force for TiN formation does not automatically prevent prior or concurrent silicidation, but in the present study, the high nitrogen partial pressure and the initial stacking of a Ti/TiN bilayer favor nitride formation kinetically before Si–Ti diffusion can proceed to completion.

The formation of Ti_5_Si_4_ and TiN rather than TiSi_2_ in the nitrogen-containing stacks is thus consistent with thermodynamically driven competition: the more negative Δ*G_f_* for TiN formation diverts Ti atoms into nitride formation before sufficient Si–Ti interdiffusion occurs to produce the Si-rich TiSi_2_ composition. However, establishing the detailed mechanism, whether TiN blocks Si transport across a diffusion barrier, whether the local Si concentration becomes depleted in the Ti layer, or a combination of factors, requires compositional profiling across the interfaces and quantitative knowledge of nitrogen partial pressure during the experiment. Bulk thermodynamic formation energies alone cannot demonstrate Si–Ti diffusion blockage or predict the quantitative distribution of phases.

However, one can resort to resistivity measurements conducted here and literature values. The Ti_5_Si_4_ nanorods resistivity of 348 μΩ·cm was measured [26]. TiN value in [16] describes a separately deposited film after N_2_ annealing. Within the adopted bulk thermodynamic description, Ti_5_Si_4_ + 6Si → 5TiSi_2_ has Δ*G_f_* = −163.87 kJ per reaction at 873 K [21]. Thus, observation of Ti_5_Si_4_ cannot be explained merely by ranking formation energies per formula unit, and local Si availability and kinetic limitations must also be considered. Table 2 supplies thermodynamic context and resistivity values.

Under high vacuum at 10^−3^ Pa, the thermodynamic and kinetic conditions favor direct C54-TiSi_2_ formation via grain boundary nucleation, yielding a low-resistivity contact. In N_2_ atmosphere, competing TiN formation thermodynamically suppresses silicidation, leading to Ti_5_Si_4_ and TiN co-formation and elevated contact resistance.

**Figure 6:**
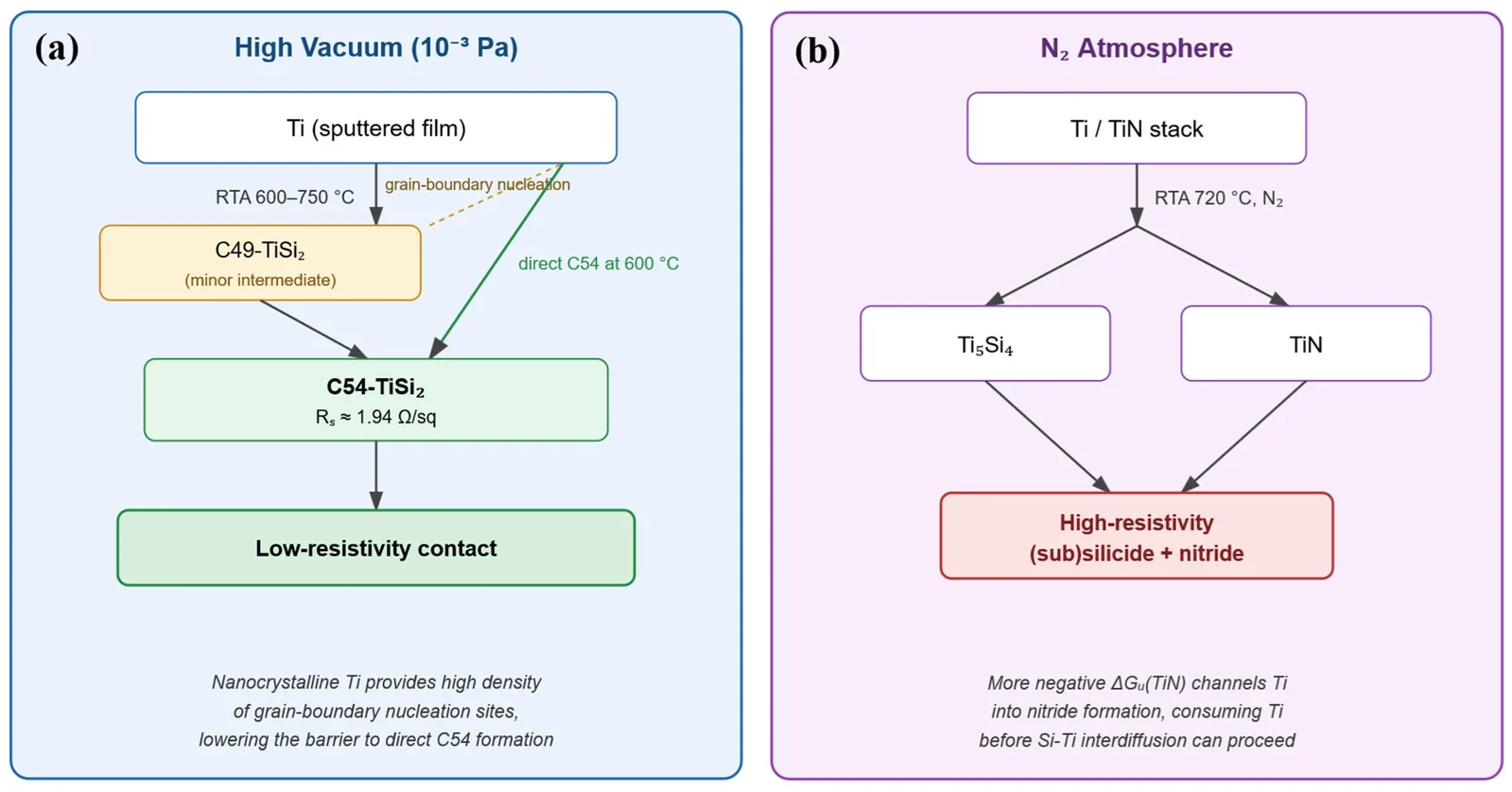
Schematic diagram of competitive phase formation pathways in the Ti/Si system under (**a**) high vacuum versus (**b**) N_2_ atmosphere.

Figure 6 provides a schematic overview of the two competing phase formation pathways identified in this study. Under high vacuum, the clean chemical environment removes the competitive driving force for nitride formation, enabling Si-Ti interdiffusion to proceed uninhibited. The nanocrystalline character of the sputtered Ti film provides a high density of grain boundary nucleation sites for C54, facilitating direct C54 formation at 600 °C without requiring the conventional intermediate C49 stage [7,10]. In contrast, the N_2_ atmosphere pathway is dominated by the much more negative Δ*G_f_* of TiN, which channels Ti atoms into nitride formation before they can react with silicon, producing the less conducting Ti_5_Si_4_ sub-silicide instead.

### 4.5. Model Validation Against Experimental Data

The combined Stefan–Boltzmann, Arrhenius diffusion, and Gibbs free energy framework provides quantitative predictions that align with experimental observations in Table 3. The direct formation of the C54-TiSi_2_ phase at 600 °C, approximately 100–150 °C below the conventionally reported C49-to-C54 transition range of 700–900 °C, is explained by the nanocrystalline nature of magnetron-sputtered Ti films. The high density of grain boundary triple junctions provides abundant heterogeneous nucleation sites with reduced activation barriers relative to homogeneous nucleation required in bulk crystalline material.

Diffusion length calculations confirm that the diffusion length of 2 μm at 873 K exceeds the 50 nm film thickness, making complete kinetic transformation feasible. This aligns precisely with the observed sharp 30-fold reduction in sheet resistance at 600 °C, confirming the conversion of metallic Ti into the highly conductive C54 phase.

The suppression of TiSi_2_ in nitrogen atmosphere is captured by the thermodynamic analysis. Table 1 shows that Δ*G_f_*(TiN) is substantially more negative (−254.43 to −240.58 kJ/mol) than Δ*G_f_*(TiSi_2_) (−171.07 to −170.39 kJ/mol), favoring nitride formation and blocking Si–Ti interdiffusion. This explains the experimental observation of Ti_5_Si_4_ and TiN phases rather than TiSi_2_ when TiN capping layers are present and annealing is performed in N_2_.

The gradual decrease in sheet resistance between 600 and 750 °C in Figure 3 is consistent with the model prediction that no new phase transition occurs across this range. Instead, the small 1.21-fold conductivity improvement results from grain growth and structural ordering within the already-formed C54 phase, as confirmed by XRD narrowing in Figure 2.

Finally, the model accounts for TiN texture development under prolonged annealing in nitrogen, where grain reorientation from (111) to (200)-preferred orientation is driven by surface energy minimization in FCC nitride films, consistent with the AFM observations of increased roughness in Figure 5.

Overall, the agreement between theoretical predictions and experimental data confirms that the combined Stefan–Boltzmann energy framework, Arrhenius diffusion kinetics, and Gibbs free energy analysis provide a reliable mechanistic explanation for phase evolution in Ti/Si systems under different annealing conditions and atmospheres. This validation strengthens confidence in applying the model to optimize processing parameters for industrial silicidation and contact formation in advanced device technologies.

## 5. Discussion

The experimental results presented in this work demonstrate that rapid thermal annealing in 10^−3^ Pa high vacuum effectively promotes the transformation of magnetron-sputtered Ti films into C54-TiSi_2_. The early formation of the C54 phase at 600 °C represents a meaningful technological advance: the conventional C49-to-C54 transition temperature is typically reported in the range of 700 to 900 °C for bulk single-crystal silicon substrates [1,4,7]. The approximately 50 to 100 °C reduction achieved here is attributable to the nanocrystalline character of the sputtered Ti films, which provide a significantly higher density of grain boundary triple junctions and thereby lower the effective nucleation barrier for C54 formation [17].

From a thermodynamic perspective, the Stefan–Boltzmann analysis quantifies the energy benefit of this lower synthesis temperature. Reducing the process temperature from 923 K to 873 K decreases the radiative energy output of the annealing equipment by approximately 20%, and relative to the conventional C49-to-C54 transition at 973 K, the savings exceed 35%. In high-throughput semiconductor manufacturing environments where hundreds of wafers are processed daily, this reduction in thermal budget translates directly to lower energy consumption and reduced thermal stress on device structures [10].

The gradual decrease in sheet resistance observed between 650 and 750 °C in Figure 3 does not correspond to a new phase transition. In this temperature range, the C54 phase is already fully formed, and the marginal conductivity improvement reflects progressive crystallite growth and reduction of grain boundary scattering within the existing C54-TiSi_2_ layer. The 1.21-fold change in R_s_ over 150 °C is consistent with grain boundary-dominated electrical transport in nanocrystalline films [2,4]. The annealing temperature of 700 °C appears optimal in terms of balancing phase purity, structural order, and minimal thermal budget.

The suppression of TiSi_2_ formation in N_2_ atmosphere is thermodynamically driven by the much more negative Gibbs free energy of TiN formation, as shown in Table 1 and Table 2. This finding is consistent with prior reports documenting the formation of TiN surface layers during RTA of Ti films in nitrogen-containing environments [9,15]. While TiN layers formed on top of TiSi_2_ are commonly exploited as diffusion barriers against Cu in SALICIDE technology [16], the present results demonstrate that a dominant N_2_ atmosphere fundamentally prevents TiSi_2_ formation altogether. The formation of Ti_5_Si_4_ as the predominant silicide phase in N_2_ atmosphere is attributed to the nitrogen-induced suppression of Si–Ti diffusion, because the kinetically favored formation of Ti–N bonds at the Ti/Si interface, combined with potential nitrogen-induced blocking of Si diffusion, prevents sufficient Ti migration to form the silicon-rich TiSi_2_ composition [9,15].

Comparison with recent work on alternative low-temperature C54 synthesis routes is instructive. Guelladress et al. demonstrated that nanosecond laser annealing of amorphous Si/Ti/amorphous Si stacks can lower the C54 formation temperature to 600 °C by generating a C40-TiSi_2_ template phase during the laser pulse [10]. The present work achieves a comparable temperature reduction through a different mechanism, namely nanocrystalline grain boundary engineering via controlled magnetron sputtering, without requiring the additional complexity of laser treatment. This approach is therefore more directly compatible with conventional RTA equipment already deployed in semiconductor fabrication lines.

The prolonged annealing duration effect observed for the TiN/Ti_5_Si_4_ system reveals an additional dimension of microstructure evolution. The fivefold increase in annealing time drives TiN grain reorientation from (111)- to (200)-preferred texture, consistent with surface energy minimization arguments for FCC nitride films [15,16]. The corresponding surface roughening observed by AFM has practical implications: excessive TiN roughness may increase contact resistance variability and reduce the reliability of metal interconnects built on top of these layers [15].

## 6. Conclusions

The following conclusions are drawn from the present investigation:

(1) Annealing Ti films in a tube furnace at residual pressures of 7 to 8 Pa results in oxidation due to trace oxygen, transforming Ti into TiO_2_ and increasing sheet resistance from 70 to 90 Ω/sq. Annealing under atmospheres of several pascals is therefore unsuitable for silicidation applications.
(2) Rapid thermal annealing of 50 nm Ti films in high vacuum (10^−3^ Pa) at 600 °C for 5 min leads directly to the formation of nanocrystalline C54-TiSi_2_, reducing sheet resistance by approximately 30-fold (from 56.15 to 1.94 Ω/sq). This confirms that the C54 phase can form at temperatures approximately 50 °C below the values typically reported for conventional single-crystal substrates.
(3) According to the Stefan–Boltzmann law, lowering the synthesis temperature from 923 K to 873 K reduces the energy consumption of industrial annealing equipment by at least 20%, representing a technologically significant gain for high-throughput semiconductor manufacturing.
(4) Annealing in N_2_ atmosphere suppresses TiSi_2_ formation. Ti_5_Si_4_ sub-silicide and TiN nanocrystals form instead, driven by the substantially more negative Gibbs free energy of TiN formation and the preferential formation of Ti-N bonds over Ti-Si bonds in nitrogen-containing environments.
(5) Extending the annealing duration in N_2_ promotes texturing of TiN films, with preferential reorientation from (111) to (200) and enhanced surface granulation as observed by AFM and XRD.
(6) The proposed thermodynamic and diffusion model, incorporating grain boundary nucleation theory, Arrhenius diffusion kinetics, and Gibbs free energy analysis, consistently explains all experimental observations and provides a predictive framework for optimizing RTA conditions in TiSi_2_ synthesis.

Overall, 10^−3^ Pa high vacuum RTA is an effective route for synthesizing low-resistivity C54-TiSi_2_ contacts on silicon by magnetron sputtering, offering both structural quality and energy-efficient processing. These findings have direct relevance to contact engineering in advanced CMOS and MEMS device fabrication.

## Figures and Tables

**Table 1:** Standard formation quantities for TiSi_2_ and TiN. TiSi_2_ values are model calculations [21], while TiN values are interpolated from JANAF [19].

T K	Δ*S_f_* J/(mol·K) TiSi_2_	Δ*S_f_* J/(mol·K) TiN	*T*Δ*S_f_* kJ/mol TiSi_2_	*T*Δ*S_f_* kJ/mol TiN	Δ*H_f_* kJ/mol TiSi_2_	Δ*H_f_* kJ/mol TiN	Δ*G_f_* kJ/mol TiSi_2_	Δ*G_f_* kJ/mol TiN
873	−4.55	−92.72	−3.97	−80.94	−175.04	−335.37	−171.07	−254.43
923	−4.55	−92.45	−4.2	−85.33	−175.04	−335.13	−170.84	−249.8
973	−4.55	−92.22	−4.43	−89.73	−175.04	−334.91	−170.61	−245.19
1023	−4.55	−92.04	−4.65	−94.15	−175.04	−334.73	−170.39	−240.58

**Table 2:** Phase properties and reported processing conditions.

Phase	Crystal Structure	Δ*G_f_* kJ/mol	R_s_ μΩ·cm	Conditions and Qualification
TiSi_2_-C54	Face-centered orthorhombic	−171.07 [21]	15–20 [10]	After 600–750 °C vacuum anneal
Ti_5_Si_4_	Tetragonal	−691.47 [21]	348 [26]	In N_2_ with TiN caps. Nanorods
TiO_2_ (rutile)	Tetragonal	−784.94 [19]	N/A	Oxygen partial pressure not reported
TiN	Face-centered cubic	−254.43 [19]	11 [16]	Observed after N_2_ annealing

**Table 3:** Comparison of model predictions with experimental observations.

Experimental Observation	Model Prediction	Agreement
C54-TiSi_2_ forms at 600 °C	Nanocrystalline Ti reduces Δ*G* via higher grain boundary density. Diffusion length exceeds film thickness at 600 °C.	Consistent
Sheet resistance drops approximately 30-fold at 600 °C	Complete Ti-to-TiSi_2_ conversion. Arrhenius model confirms kinetic feasibility.	Consistent
N_2_ atmosphere produces Ti_5_Si_4_ and TiN, not TiSi_2_	Δ*G_f_*(TiN) much more negative than *ΔG_f_*(TiSi_2_). Ti-N bond blocks Si diffusion.	Consistent
Gradual R_s_ decrease from 600 to 750 °C	C54 grain growth and structural ordering. No new phase transition.	Consistent
TiN texture development with prolonged N_2_ annealing	Grain growth and reorientation driven by surface energy minimization in TiN.	Consistent

## Data Availability

The data supporting the findings of this study are available from the corresponding authors upon reasonable request.
